# Collective Impact Approaches to Promoting Community Health and Wellbeing in a Regional Township: Learnings for Integrated Care

**DOI:** 10.5334/ijic.5617

**Published:** 2021-06-21

**Authors:** Alan Hayes, Margaret Freestone, Jamin Day, Hazel Dalton, David Perkins

**Affiliations:** 1 Family Action Centre, University of Newcastle, Australia; 2 Centre for Rural and Remote Mental Health, University of Newcastle, Australia

**Keywords:** health and wellbeing, integrated care, collective impact

## Abstract

This Perspective Paper explores the challenges of implementing local initiatives guided by the tenets of the Collective Impact (CI) approach. As such, it draws implications of CI for integrated health and social care efforts to improve and sustain health and social outcomes within a community-wide context, based on our efforts to deploy a CI intervention in the regional town of Muswellbrook, New South Wales (NSW) Australia. A program of health and wellbeing activities providing mental health and wellness messages and activities was implemented in the township over 2 years by the Family Action Centre (FAC), University of Newcastle, Australia. A key takeaway was the importance of authentic community engagement and active involvement as opposed to mere consultation.

## Context for this Reflection

The current pandemic highlights the prime importance of mobilising integrated and coordinated action to address a wickedly complex problem. There are parallels that can be drawn from the key elements of global responses to the current situation, which have incorporated public health campaigns to educate, engage and mobilise communities; preventative measures, including behaviour changes such as social distancing, improved hygiene, personal protective equipment use, including mask wearing; along with intervention approaches such as quarantine, self-isolation and intensive care. These responses have been underpinned and framed by concerted scientific action to understand the problem (i.e., the disease), discover preventative solutions (i.e., vaccines) and advance innovative treatment options.

Whether it is at the global pandemic scale or at the local level, effective solutions to complex problems require collective action to achieve impact. The Collective Impact (CI) approach provides a framework to integrate resources and address priority problems.

## Brief description of the focus

CI seeks to engage and mobilise stakeholders in a community to address complex health, social and economic challenges by a) developing a common agenda to identify and prioritise the problem(s) to be addressed; b) agreeing on what success should look like and how it would be measured; c) ensuring that activities are mutually reinforcing; d) facilitating continuous communication; and most importantly e) creating a backbone structure to support project and data management responsibilities [1]. As such, these are elements that also resonate with the logic of integrated health and social care.

In 2016, Muswellbrook was identified by the NSW Government as a community confronting a range of challenges, that warranted innovative, coordinated prevention and early intervention efforts. Muswellbrook is a township of over 16,000 people in the Upper Hunter region of NSW. The main industries are coal mining, power generation and agriculture, including equine enterprises [2]. The Government’s concern was that, despite many local assets and strengths, the community faced health, wellbeing and social challenges. These included the impending impacts of changing economic and social conditions, given likely local and global changes in the major industry, coal mining.

A community place-based Working Group was created to address these issues. It included representatives from the relevant NSW government community services, education, housing, and transport departments; the Shire Council; the Indigenous community; and the local social housing provider. The Working Group invited academics from the FAC to collaborate in addressing these complex issues by employing an approach informed by CI and focusing on positive strategies to improve the health and wellbeing of children and families in the Muswellbrook community.

The group adopted a prominent Australian framework for Collective Impact encompassed by the acronym **CREATE**. The CREATE model employs a program logic structured around its acronym: **C**ollaborative, to involve many stakeholders; **R**elationship-driven, to build trust and local ownership; **E**arly in the pathway, to prioritise prevention; **A**ccountable to stakeholders; **T**raining, to build local capability; and **E**vidence-driven, to demonstrate measurable change in outcomes [3].

Despite adopting key elements of the CREATE approach, and re-branding itself as the CREATE Change Coalition, the group struggled to achieve consensus on the priority problems and actions to address these, given the range of possible health, wellbeing and social challenges confronting the community. Among other key impediments to progress were the Coalition’s inability to develop a community engagement strategy; to include grassroots community representation; and to provide the resources required to support the initiatives essential to achieving collective action and sustainable solutions. The lack of meaningful engagement in the process significantly limited the effectiveness of the Coalition’s efforts.

In the face of these challenges, the FAC designed and implemented an integrated collaborative program within the community — Muswellbrook Healthy & Well (MH&W) [4]. Funding from philanthropic foundations enabled the appointment in June 2018 of a local coordinator for the initiative.

With additional support from the University’s Centre for Rural and Remote Mental Health (CRRMH) [5] MH&W was able to deploy the Act-Belong-Commit program (A-B-C), a Western Australian population-based health promotion strategy designed to address community health and wellbeing needs and encourage behavioural change via a range of community led initiatives [6]. A-B-C aims to increase individuals’ mental health literacy and incorporates engaging health promotion activities via participation in community groups.

In addition, the FAC’s program *Our Health Rules!* (OHR!) was deployed as a locally relevant nutrition and dietetics program, given the high rates of obesity and diabetes within the Muswellbrook community. Through nutrition education, cooking and healthier lifestyle programs, OHR! aims to prevent chronic disease by enhancing nutrition knowledge and skills in order to improve the health and wellbeing of families and their children [7].

## Discussion & Reflection

Across the 2 years of the MH&W program almost 4,500 participants have been involved in the various integrated aspects of the program in the township. Consultation within the community and with more than 80 stakeholders from local organisations resulted in the co-operative co-design and implementation of locally appropriate activities. Community engagement efforts started with open days to provide public information on the program initiatives, to raise awareness and identify activities that would mobilise community involvement in MH&W.

The range of different types of activities that have been developed to cover different community ages and interests provides evidence of the value of harnessing CI to identify local priorities to facilitate and sustain community engagement. Examples of activities include a ‘photovoice’ competition, designed to encourage positive health behaviours, physical activity and family engagement through photography and personal reflection [8]; team-based physical activities such as the ‘10,000 Steps’ challenges; along with the OHR! community nutrition and cooking classes. These have engaged participants across all age groups – from small children, school students and their parents, through to older community members. In addition, MH&W has also coordinated activities for specific groups including people with a disability and members of the Indigenous community. The use of web and social media resources were important in the establishment and early development of MH&W, and have proved invaluable in continuing community collective action during the COVID-19 lockdown, overcoming the constraints that it has created.

## What have we learnt?

Implementation of MH&W has highlighted the gulf between rhetoric and reality in achievement of collective action to effect sustainable change. This project, along with others worldwide [9], shows that authentic community engagement, co-design and respectful collaboration are the keys to achieving consensus on an agreed focus regarding the “problem(s)” to be targeted. They provide the foundations for mobilising collective action and designing programs that are likely to promote and sustain active community participation.

Integrated responses by various community groups were needed to respond to the priority issue of strengthening community health and wellbeing. Broad community support is essential to achieving program aims and outcomes. To make place-based initiatives sustainable, however, is “easier said than done”. Consistent with the core tenets of CI, success and sustainability require ongoing local stakeholder commitment to the ideals or messages of the initiative. In Muswellbrook, the local Steering Committee for MH&W has been vital to achieving the successes to date, but in the longer term will need to identify a backbone organisation other than the University.

The challenge going forward is for the local community to own the program, so they are not perpetually reliant on the provider. The ‘caravan of government interest’, funding and programs moves all too quickly to address the next policy priority and community involvement can wane as trust is eroded, to be replaced by an enervating cynicism.

For a program to be sustainable it needs to be strengths-based [10]. The CI approach adopted by MH&W utilized the strengths-based ‘bottom-up’ acceptance and genuine community consultation rather than ‘top-down’ enablement. This initiative was an instructive example of a successful community intervention that provides a platform for integration of approaches to address local health and wellbeing priorities. The benefits and challenges realised in this local CI community program could be transferred, across nations, to any community focused on integrating targeted local health and social care initiatives.

Like the worldwide response to COVID-19, the learnings from MH&W reinforce the value of a clear focus on the priority problem(s); establishing locally owned preventative efforts underpinned by evidence-based approaches; and co-design of innovative solutions through effective integration of the expertise of key local stakeholders, including professionals, service providers and community members. In this way, integrated health and social care can progress powerful long-term solutions for contemporary ‘wicked problems’ [11].
